# An Abdominal Case of Interest

**Published:** 1909-12-18

**Authors:** 


### AN ABDOMINAL CASE OF INTEREST.

The patient was a man aged 37, a stoker by trade. His family history was unimportant. During the past year he had from time to time suffered from pains of a "crampy" nature in the abdomen; but these attacks neither caused sickness nor compelled the patient to remain in bed. Six days before his admission to the hospital the patient was suddenly attacked with severe abdominal pain. He was at work stoking at the time, and as he had just eaten a green apple he attributed the symptom to this. The pain did not pass off, however, and he was taken home and put to bed. He became so collapsed that a doctor was sent for. Hot flannels and opium stupes were applied to the abdomen, but the patient's condition did not improve. The pains increased, but they were restricted almost entirely to the left side and upper part of the abdomen. During the first twenty-four hours of the illness the patient had vomited six times, and the vomiting recurred thrice on the second day, and once on the fifth. There had been absolute constipation for six days. The vomit was greenish in colour, and apparently at no time was it of a fæcal character. Notwithstanding the persistent vomiting and constipation, fluid food had been taken regularly and well.

On admission, the pulse-rate was 130, the respiration rate 29, and the temperature 101° F. The general appearance of the patient was that of a man in distress from pain. He was strong and muscular; his eyes were not sunken, the colour of his face and lips was good, and the tongue, though slightly coated with fur, was moist. In fact, the patient did not give one the idea of being *in extremis*. On superficial examination he was obviously sensible, and quite clear in his answers to questions.

The abdomen was very much distended, and moved only slightly with respiration. On each side of the abdomen there was a ridge over the site of the ascending and descending colon. Peristalsis could be seen at intervals. On palpation the abdominal wall was found to be uniformly rigid and distended. At no one place did there seem to be more resistance than at another. On percussion there was tympanitic resonance all over the front, but in the flanks there was distinct impairment of note when the patient lay upon his back. When he rolled upon one side, the other or uppermost became fully resonant at once. The liver dulness was normal. The tongue had a brown fur, and was not dry, but flabby. Rectal examination was negative.

Two hours later the pulse ran up to 140 per minute, and the temperature came down to 100.4° F. There was no vomiting during this time, but it was clear that the patient was getting worse. There was an operation, but the man died six hours after it.

Fat necrosis in the omentum and elsewhere in the abdomen was found at the time of operation. The diagnosis was thus clearly one of acute pancreatitis.

